# Arsenic trioxide (As_2_O_3_) as a maintenance therapy for adult T cell leukemia/lymphoma

**DOI:** 10.1186/s12977-020-0513-y

**Published:** 2020-03-21

**Authors:** Ambroise Marçais, Lucy Cook, Aviva Witkover, Vahid Asnafi, Véronique Avettand-Fenoel, Richard Delarue, Morgane Cheminant, David Sibon, Laurent Frenzel, Hugues de Thé, Charles R. M. Bangham, Ali Bazarbachi, Olivier Hermine, Felipe Suarez

**Affiliations:** 1 Service d’Hématologie Adultes, Institut Imagine, Hôpital Universitaire Necker Enfants Malades, APHP, Université de Paris, 149-161 rue de Sèvres, 75743 Paris Cedex 15, France; 2 Section of Virology, Wright-Fleming Institute, Imperial College, London, UK; 3 Laboratoire d’onco-hématologie, Institut Necker-Enfants Malades, INSERM U1151, Hôpital Universitaire Necker Enfants Malades, APHP, Université de Paris, Paris, France; 4 Laboratoire de Microbiologie, Hôpital Universitaire Necker Enfants Malades, APHP, Université de Paris, Paris, France; 5 Institut Universitaire d’Hématologie, Hôpital St. Louis, Paris, France; 6 Department of Internal Medicine, American University of Beirut, Beirut, Lebanon

**Keywords:** Arsenic trioxide, ATL

## Abstract

**Background:**

Adult T-cell leukemia-lymphoma (ATL) is an aggressive mature lymphoid proliferation associated with poor prognosis. Standard of care includes chemotherapy and/or the combination of zidovudine and interferon-alpha. However, most patients experience relapse less than 6 months after diagnosis. Allogeneic stem cell transplantation is the only curative treatment, but is only feasible in a minority of cases. We previously showed in a mouse model that Arsenic trioxide (As_2_O_3_) targets ATL leukemia initiating cells.

**Results:**

As_2_O_3_ consolidation was given in 9 patients with ATL (lymphoma n = 4; acute n = 2; and indolent n = 3), who were in complete (n = 4) and partial (n = 3) remission, in stable (n = 1) and in progressive (n = 1) disease. Patients received up to 8 weeks of As_2_O_3_ at the dose of 0.15 mg/kg/day intravenously in combination with zidovudine and interferon-alpha. One patient in progression died rapidly. Of the remaining eight patients, three with indolent ATL subtype showed overall survivals of 48, 53 and 97 months, and duration of response to As_2_O_3_ of 22, 25 and 73 months. The other 5 patients with aggressive ATL subtype had median OS of 36 months and a median duration of response of 10 months. Side effects were mostly hematological and cutaneous (one grade 3) and reversible with dose reduction of AZT/IFN and/or As_2_O_3_ discontinuation. The virus integration analysis revealed the regression of the predominant malignant clone in one patient with a chronic subtype.

**Conclusion:**

These results suggest that consolidation with As_2_O_3_ could be an option for patients with ATL in response after induction therapy and who are not eligible for allogeneic stem cell transplantation.

## Background

Adult T-cell leukemia-lymphoma (ATL) is an aggressive and mature lymphoid proliferation associated with the human T cell leukemia virus type 1 (HTLV-1) [1, 2]. ATL patients have a heterogeneous presentation and the Shimoyama classification divides the disease into four major subtypes, from indolent, slowly progressive disease (smouldering and chronic) to aggressive and life-threatening disease (lymphoma and acute) [3]. All are characterized by a dismal long-term prognosis and a low median survival rate, of 8 months, 10 months, 31 months and 55 months for the acute, lymphoma, chronic and smouldering subtypes respectively [4].

Although chemotherapy combinations improve the response rate in aggressive ATL subtype, the overall survival remains poor. Patients with indolent ATL subtype have a better prognosis but long-term survival is also poor with a watchful-waiting policy or with chemotherapy [4]. The combination of zidovudine (AZT) and interferon-alpha (IFN) may induce long term response in indolent and a small proportion of acute type [5]. Allogeneic stem cell transplantation is the only curative treatment in responding patients but its use is limited to a minority of patients [6]. The anti-CCR4 antibody mogamulizumab showed interesting results in relapsed patients, but a randomized trial in newly diagnosed ATL showed no benefit of its addition to chemotherapy in term of progression-free survival (PFS) and overall survival (OS), despite an increase response rate [7, 8].

In prior ex vivo studies, we showed that arsenic trioxide synergizes with IFN to selectively induce ATL cell apoptosis through the degradation by the proteasome of the oncoprotein Tax [9, 10]. This combination showed some signals of efficacy but a low rate of response in relapsed or refractory ATL patients [11]. A pilot study reported 100% response rate including 70% complete remission in newly diagnosed chronic ATL patients treated with the combination of arsenic, IFN and AZT [12]. We recently showed that this combination cures ATL developed in Tax-transgenic mice through selective targeting of leukemia-initiating cell (LIC) activity [13], suggesting that the best use of arsenic and IFN may be as a consolidation or maintenance therapy. Thus, we performed a retrospective study analyzing the outcome of patients treated with the combination of arsenic trioxide and AZT/IFN as consolidation after induction therapy with chemotherapy or antiviral therapy.

## Patients and methods

### Patients and diagnosis criteria

This retrospective study included nine newly diagnosed, previously untreated ATL patients. Patients’ characteristics are described in Table 1. This study was approved by the local ethic committee (CNIL: number 1692254 and CPP IRB registration number 000001072). TP53 status was evaluated in seven patients by a functional assay as previously described [14].

**Table 1:** Patient’s characteristics

Patient	Sex	Age at diagnosis	Clinical subtype	p53 activity	First line treatment	Interval between induction therapy and As_2_O_3_ consolidation (months)	Disease status at time/after of A_2_So_3_	Treatment duration (weeks)	OS since diagnosis (months)	Death	OS since arsenic (months)	Duration of response since As_2_O_3_ (months)	Progression
ATL 6	M	51.6	Chronic	F	AZT-IFN-VP16	24.4	SD/SD	4	53	Yes	28	22	Yes
ATL 9	M	50.6	Chronic	F	CHOP like	20.1	CR/CR	6	48	Yes	28	25	Yes
ATL 11	M	27.7	Chronic	F	LSG 15 + AZT-IFN	13.2	VGPR/CR	6	97	Yes	83	73	Yes^a^
ATL 7	F	59.8	Acute	F	CHOP like	6.2	PD/PD	4	8	Yes	1	NA	Yes
ATL 14	F	35.1	Acute	NF	Alemtuzumab-CHOP	2.2	CR/CR	4	12	Yes	10	5	Yes
ATL 43	F	66.3	Lymphoma	F	CHOP like	6.3	CR/CR	8	19	Yes	12	5	Yes
ATL 44	F	54.8	Lymphoma	F	CHOP like	3.8	VGPR/CR	8	36	Yes	32	29	Yes
ATL 64	M	63.1	Lymphoma	ND	CHOP/DHAOx	13	CR/CR	8	65	No	51	51	No
ATL 65	M	54.4	Lymphoma	ND	CHOP	4	PR/PR	16	41	Yes	36	10	Yes

M, male; F, female; CHOP, cyclcophosphamide, doxorubicine, oncovin, prednisone; DHAOx, dexamethasone, aracytine high dose, oxaliplatine; CR, complete response; PR, partial response; VGPR, very good partial response; SD, stable disease; PD, progressive disease

F, functional; NF, non functional (this patient was found to have a p.V274A variant of TP53)

^a^Clone switch

### Treatment schedule

After induction with chemotherapy (mainly anthracyclin-based regimen including LSG and CHOP like regimen) and/or AZT/IFN, patients received up to 8 weeks of arsenic at the dose of 0.15 mg/kg/day intravenously (Table 1) in combination with oral zidovudine (AZT; 600 mg/day) and subcutaneous recombinant IFN (Roferon, ROCHE^®^ 3 millions/day) or pegylated IFN (PEG-IFN Viraferon MSD^®^ 1.5 µg/kg/week). During the consolidation phase, patients could receive only IFN/AZT combination between the arsenic infusions.

In case of hematological toxicity, growth factors such as GCSF or EPO were used, or the antiviral therapy dose was reduced *as per* physician choice.

### Response criteria and overall survival

Response evaluation was performed according to consensus published criteria [15]. Overall survival (OS) was defined as the period between initiation of treatment and the date of death or last follow-up.

### HTLV-I proviral load and clonality assay

DNA extraction was done using Invitrogen kit (QiAmp or blood and cell’s DNA extraction kit) and performed according to manufacturer’s instructions. HTLV DNA was quantified by real-time PCR in the pX region as previously described [16]. High-throughput sequencing for the genome-wide identification and quantification of proviral integration sites was performed as previously described [17].

## Results and discussion

At time of As_2_O_3_ initiation, four patients were in CR, two in VGPR, one in PR, one had stable disease and one progressive disease. The consolidation therapy with As_2_O_3_, IFN and AZT was administered for a median period of 6 months (2–24 months) after completion of first-line induction treatment (13 months when ATL7 is excluded). Side effects were mostly hematologic and manageable with discontinuation of AZT/IFN or addition of growth factors. Four patients experienced cutaneous side effects, including one grade 3, which were all reversible on discontinuation of As_2_O_3_ (Additional file 1: Table S1). In addition to these objective toxicities, most patients experienced severe fatigue especially during the last weeks of As_2_O_3_ therapy, which was rapidly reversible after As_2_O_3_ discontinuation.

The patient with progressive disease (acute type) at time of arsenic initiation did not respond to As_2_O_3_/AZT/IFN consolidation and died rapidly. The median duration of response in the other 8 patients was 24 months (5–73 months) and 39 months (7–86 months) from initiation of As_2_O_3_/AZT/IFN consolidation and from initiation of first line treatment, respectively. Median OS was 28 months (1–70 months) and 42 months (8–97 months) from As_2_O_3_/AZT/IFN consolidation start and initial diagnosis, respectively (Table 1). Two patients experienced a prolonged survival (Chronic n = 1, Lymphoma n = 1). One patient with a lymphoma subtype remained in remission 51 months from arsenic initiation and 64 months from diagnosis. The other patient with a chronic subtype remained in remission 86 months after Arsenic initiation and 97 months after diagnosis before relapsing with a new tumoral clone as described below. The three indolent ATLs had an OS of 48, 53 and 97 months and duration of response to As_2_O_3_ of 22, 25 and 73 months. The six aggressive ATLs had a median OS of 27,5 months (range 8–65 months) and a median duration of response to As_2_O_3_ of 10 months (5–55 months).

**Fig. 1:**
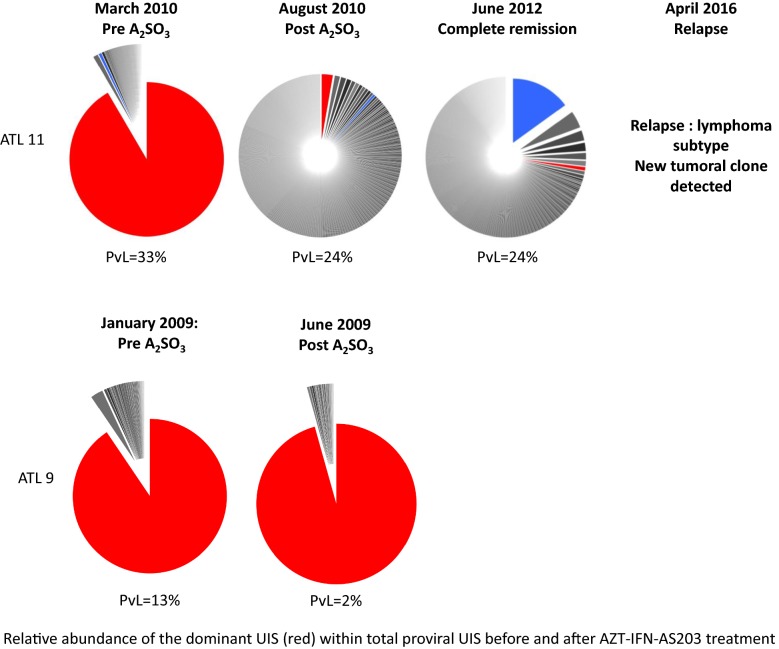
Virus clonality architecture timeline. Responding (ATL 11) and resistant patients (ATL 9)

Longitudinal analysis of the HTLV-I proviral load (PvL) revealed no difference during treatment except in one patient (ATL 14) with acute ATL, who showed a dramatic decrease of proviral load after chemotherapy. Viral integration clonality analysis was assessed in 2 patients. One patient (ATL 11) who had a normal lymphocyte count but with an excess of phenotypically abnormal T-cells and one dominant clone representing 92% of infected cells exhibited 1 month after As_2_O_3_ treatment a regression of the predominant malignant clone and restoration of an oligoclonal architecture, both in proportion and in absolute count, while the proviral load remained stable. Interestingly, this patient remained in remission 86 months after initiation of arsenic and 97 months from diagnosis but finally relapsed with a different clone, as demonstrated by the finding of a different TCR rearrangement as previously published [18]. In contrast, another patient (ATL 9), with a chronic subtype initially treated with chemotherapy had a normal lymphocyte count with an excess of abnormal phenotype T cells with one dominant clone that represented 91% of infected cells, which remained unchanged after completion of As_2_O_3_ treatment. This patient progressed to an acute subtype 2 years later and died (Fig. 1).

Taken together, as predicted by our mice model and our previous clinical study with the triple induction with As_2_O_3_/AZT/IFN in chronic ATL, this retrospective clinical analysis shows that As_2_O_3_ consolidation in combination with low-dose AZT/IFN maintenance may enhance long-term disease control also in ATL lymphoma with moderate side effects [12, 13]. In addition, although based on a small number, our data suggest that sequential analysis of proviral load and architecture of the virus clonality could serve as a good surrogate marker of long-term response rather than the viral load and lymphocyte count.

However, despite prolonged responses in some cases, most patients ultimately relapsed, suggesting that one cycle of arsenic consolidation may not be sufficient. Future trials are warranted to investigate whether or not multiple cycles of arsenic consolidation are needed in ATL to prevent relapses.

## Supplementary information

**Additional file 1: Table S1.** Toxicity.

## Data Availability

Not applicable.

## Abbreviations

ATL: Adult T-cell leukemia-lymphoma
HTLV-1: Human T cell leukemia virus type 1
AZT: Zidovudine
INF: Interferon-alpha
PFS: Progression free survival
OS: Overall survival
LIC: Leukemic initiating cells
WT: Wild type
PEG-INF: Pegylated interferon
TCR: T-cell receptor
