# Supplementary material for: Arsenic trioxide (As2O3) as a maintenance therapy for adult T cell leukemia/lymphoma
Source: Retrovirology. 2020 Mar 21;17:5. doi: 10.1186/s12977-020-0513-y (PMC7085150; doi:10.1186/s12977-020-0513-y)
Supplement: Supplementary file 1 — Additional file 1: Table S1. Toxicity. [file 12977_2020_513_MOESM1_ESM.docx]

**Additional Table S1: Toxicity**

| Patient | Anemia | Thrombocytopenia | Neutropenia | Cutaneous | Other |
| --- | --- | --- | --- | --- | --- |
| ATL 6 | 3 | 0 | 3 | 2* | fever |
| ATL 7 | 1 | 2 | 3 | no | no |
| ATL 9 | 3 | 0 | 0 | 2* | asthenia |
| ATL 11 | 2 | 2 | 3 | **3** | no |
| ATL 14 | NA | NA | NA | 2* | no |
| ATL 43 | 2 | 3 | 3 | no | asthenia |
| ATL 44 | 2 | 2 | 3 | no | asthenia |
| ATL 64 | 2 | 3 | 3 | no | asthenia |
| ATL 65 | NA | NA | NA | no | asthenia |

*Hand and foot
